# Semiochemical oviposition cues to control *Aedes aegypti* gravid females: state of the art and proposed framework for their validation

**DOI:** 10.1186/s13071-022-05337-0

**Published:** 2022-06-25

**Authors:** Margaux Mulatier, Antoine Boullis, Anubis Vega-Rúa

**Affiliations:** 1 Laboratory of Vector Control Research, Institute Pasteur of Guadeloupe, Lieu-dit Morne Jolivière, 97139 Les Abymes, Guadeloupe France; 2 TERRA, Gembloux Agro-Bio Tech, University of Liège, Avenue de la Faculté 2B, 5030 Gembloux, Belgium

**Keywords:** *Aedes aegypti*, Gravid, Oviposition, Odours, Attractants, Deterrents, Laboratory validation, Field validation, Methodology

## Abstract

In the fight against mosquito-borne diseases, odour-based lures targeting gravid females represent a promising alternative to conventional tools for both reducing mosquito populations and monitoring pathogen transmission. To be sustainable and effective, they are expected to use semiochemicals that act specifically against the targeted vector species. In control programmes directed against *Aedes aegypti*, several candidates of different origins (conspecifics, plants) have already been identified as potential oviposition attractants or repellents in laboratory experiments. However, few of these candidates have received validation in field experiments, studies depicting the active molecules and their mode of perception are still scarce, and there are several methodological challenges (i.e. lack of standardization, differences in oviposition index interpretation and use) that should be addressed to ensure a better reproducibility and accelerate the validation of candidates. In this review, we address the state of the art of the compounds identified as potential candidates for trap development against *Ae. aegypti* and their level of validation. We also offer a critical methodological analysis, highlight remaining gaps and research priorities, and propose a workflow to validate these candidates and to increase the panel of odours available to specifically trap *Ae. aegypti*.

**Graphical Abstract:**

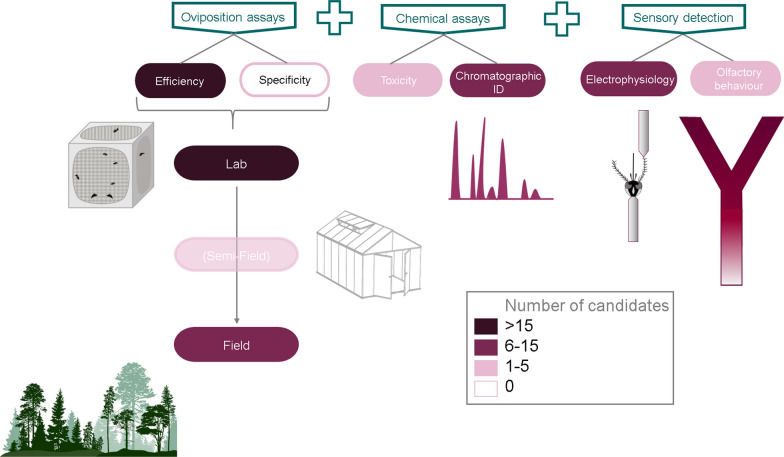

## Background

*Aedes aegypti* is a major vector of arboviruses worldwide. For decades, vector control programmes against this species have relied on the massive use of insecticides. Because the use of these methods is threatened by their detrimental side effects [1, 2], developing alternative and sustainable control strategies is imperative for the resilience of vector control [3, 4]. Such tools must be cost-effective, respectful of the environment and human health, exhibit a species-specific targeting and minimize selective pressure on the populations in order to delay the spread of resistance or adaptive mechanisms [5]. So far, several alternative tools have been developed to replace insecticides [4]. Among them, attract-and-kill systems are promising for reducing mosquito population densities and the subsequent risk of pathogen transmission, with limited impact on the non-target organisms and on the environment. Mosquito physiological stages targeted in such traps can be either host-seeking or gravid females [6].

Traps for capturing and killing gravid females (gravitraps) and/or their progeny (ovitraps) can be a significant component of integrated control programmes. First, reaching mosquito females before they have the chance to lay eggs would prevent them from producing new progeny, and, supposedly, will directly affect population densities [7]. Also, from an epidemiological point of view, gravid females have had at least one previous blood meal and are thus likely to be infected with arboviruses, which makes them logical targets for both surveillance and control.

Gravitraps were first developed for monitoring mosquito populations. They allow us to characterize many entomological indicators such as the diversity and abundance and the age structure [8, 9], but also to perform arboviral screening and to assess the infection rate of a given population [10–12]. Compared with larval sampling, traps for gravid females offer the advantage of providing data on adult vectors, as well as being more sensitive and viable than larval census for sampling species like *Ae. aegypti* [13, 14]. Gravitraps are also easy to implement and can be handcrafted, thus being a cost-effective option. Gravitraps can also be coupled with rapid diagnostic tests to provide the community with early information about transmission in a given area [15], ultimately allowing public health decision-makers to identify the priority zones for intervention. Besides surveillance, traps that attract and kill mosquitoes might help to reduce mosquito abundance locally. By targeting females that are likely to harbour pathogens, these traps would also allow selective sampling of the older females responsible for transmission, therefore reducing the incidence of an outbreak [7]. Their efficacy in reducing *Ae. aegypti* populations has been confirmed at a communal scale in areas of several countries such as Australia, Puerto Rico, and Brazil [16–18]. In some trials, epidemiological indicators confirmed a correlation between gravitraps and reduction of chikungunya incidence [10, 19]. Traps currently distributed for public use such as AGO (BioCare^®^) and BG-GAT (Biogents^®^) have shown efficacy in attracting and capturing *Aedes* mosquitoes [20, 21]. These traps respectively use an adhesive panel and a residual surface spray insecticide that can be replaced with an insecticide-free killing agent such as canola oil or adhesive sticky cards [22], which makes it possible to reach insecticide-resistant females that are no longer targeted by conventional insecticide-based tools.

Despite their potential for controlling vector mosquitoes, some drawbacks limit their full integration in global-scale programmes. First, BG-GAT and AGO are respectively composed of alfalfa and hay infusions [20, 21], attractants whose lack of specificity to *Ae. aegypti* might induce a strong impact on non-target organisms [23], therefore impeding their use as sustainable tools. Moreover, *Ae. aegypti* displays a particular oviposition behaviour, consisting in scattering egg batches among several oviposition sources to minimize the risk of reproductive failure [24, 25]. This “skip-oviposition” strategy implies that the attractiveness of a trap must be significant as compared with naturally attractive breeding sites, in order to kill females at their first attempt to lay eggs. Therefore, in the context of the development of cost-effective and long-lasting traps that target gravid *Ae. aegypti*, there is a need for identifying odours that are specifically active against this vector species, i.e. that are biologically relevant for *Ae. aegypti* gravid females and induce an oviposition behaviour [5, 26]. These last years, many chemical lures have been tested in the laboratory for their potential in mediating *Ae. aegypti* gravid female oviposition. However, whereas several candidates have been evidenced as potential oviposition mediators under laboratory experiments, few have received validation under field assays, and studies aiming at characterizing their chemical composition and sensory perception remain too scarce. This lack of knowledge strongly limits the panel of compounds available for the implementation of odour-based lures. Hence, the validation of the potential candidates already identified is urgently needed to provide a comprehensive description of the available semiochemical cues and offer opportunities for the design of highly effective and species-specific control and surveillance tools against *Ae*. *aegypti*.

In this review, we document the advances in the development of odour-based traps directed against gravid *Ae. aegypti* females and propose guidelines for identifying and/or validating potential semiochemical oviposition cues against this mosquito species. More specifically, we (i) address the state of the art for the compounds identified as potential candidates, their chemical identification and their level of validation, (ii) describe the compounds for which the sensory perception has been investigated, (iii) present the most promising compounds, (iv) identify critical methodological gaps and (v) recommend a line up for the design of experimental protocols to obtain robust and rigorous data, adapted to *Ae. aegypti* and unaffected by skip-oviposition.

### Literature search and data extraction

Our bibliographic database was elaborated by searching published articles containing different combinations of the terms “*Aedes aegypti*” AND “oviposition” OR “gravid” OR “cues” OR “trap” OR “ovitrap” OR “gravitrap” OR “behaviour” OR “electrophysiology”. Literature searches were conducted between March 2020 and May 2021 using PubMed, Web of Science and Google Scholar. The examination of the pertinence and quality of the bibliography identified allowed the inclusion of 96 studies in this review. Oviposition behaviour, oviposition attractants for other mosquito species and attractants for host-seeking females have been described in detail in recent reviews [6, 27–29] and were therefore not included in our study.

### Metrics for assessing oviposition preferences

When searching for cues that are preferred or avoided by gravid females, experiments are generally performed in dual-choice bioassays, where mosquitoes are offered either the tested compound or the control as substrates for oviposition. Females are tested by groups of varying size, in cages (laboratory experiments) or experimental chambers (semi-field experiments). Field experiments involve placing several traps in a given area, where the recorded parameter is either the number of eggs received or the number of females caught. Field experiments are generally conducted to compare the efficacy of a candidate to water [30–33] or to existing attractants [31] and can also help in improving the efficacy of commercial traps [20, 31, 34, 35]. Egg-count is conventionally the recorded parameter in these bioassays, where the number of eggs laid per female is estimated by dividing the total number of eggs counted by the number of females introduced into the cage. The effect on oviposition is calculated as the oviposition activity index (OAI), initially described by Kramer & Mulla [36] and calculated as follows: OAI = (NT – NS) / (NT + NS), where NT and NS denote the mean number of eggs laid in the treatment and control, respectively. OAI values fall within +1 to −1, with positive values indicating oviposition stimulation/attraction, and negative values indicating repellence/deterrence. Results are generally presented by giving both the OAI and the corresponding *P*-value (obtained with Kruskal–Wallis tests, *P* < 0.05). In the present work, all the OAIs cited are associated with a significant *P*-value.

### Origin of oviposition signals

Olfactory and gustatory cues are crucial signals that help gravid *Ae. aegypti* females detect and select suitable oviposition sites within their environment. They provide information about food availability for offspring, potential competition between conspecifics and heterospecifics, and predation risk (reviewed in [37]). Site-finding starts from a wide distance (more than a few metres), where visual and long-range olfactory (i.e. highly volatile) cues drive the choice of one site over another [38, 39]. Then, at a shorter distance (from a few decimetres until contact with the odour source), semi-volatile and tactile chemical cues allow females to perform the last evaluation and choose whether to accept or reject a breeding site [39] (reviewed in [6, 27–29]).

The chemical signals may originate from different organic sources, such as conspecifics, heterospecifics, plant infusions or bacterial metabolism [27]. They can be perceived by females individually or in association. For example, the presence of *Ae. aegypti* immature stages in the water is a strong determinant of female oviposition choice, and may influence the acceptance of a breeding site following a density-dependent response [40–45]. The acceptance generally follows a trade-off between suitable conditions for offspring development and overcrowding or potentially detrimental conditions and is based on factors such as larval nutritional state, infection status, and density. Hence, identifying the chemical signature of immature stages is of great interest because it could provide good chemical candidates involved in pheromonal communication that can be used to implement traps that specifically target *Ae. aegypti*. Few studies have already described compounds from immature stages, all using solvent extraction methods to extract organic compounds. Chemical compounds have been identified in egg extracts*,* including short-chain fatty acids such as caproic acid (C6:0) [46], as well as higher fatty acids of carbon chain length from C12 to C18, such as dodecanoic acid (C12:0), tetradecanoic acid (C14:0), hexadecanoic acid (C16:0), Z-9 hexadecenoic acid (C16:1n7), and their methyl esters, along with a lactone [47]. On the other hand, a hydrocarbon, *n-*heneicosane, has been isolated from the cuticle of *Ae. aegypti* larvae [48]. Also, a recent study identified 15 compounds (including eight fatty acids, two corresponding methyl esters and one lactone) in larval and pupal extracts, which represents to date the widest variety of larval compounds identified [49]. Despite the variety of chemical signals described in these studies, there are no data available about volatile compounds emitted from water containing immature stages of *Aedes* mosquitoes. The sampling of headspace from larval rearing water would be a good technique for detecting volatile cues that possibly trigger oviposition behaviour of *Ae. aegypti* gravid females at a wider distance, as was observed with *Anopheles* mosquitoes [50, 51].

The presence of heterospecifics within a potential breeding site, such as predators (i.e. the larvivorous fish *Betta splendens,* the copepod *Mesocyclops longisetus* and the mosquito *Toxorhynchites theobaldi*) [52–54], parasites (*i.e*. the trematode *Plagiorchis elegans*) [42, 55], or competitors (i.e. *Aedes albopictus*) [44, 56] can also influence the ovipositional responses of *Ae. aegypti*. However, whereas some chemicals originating from predators have been characterized [53], their role in the oviposition behaviour remains to be tested in laboratory. Also, the presence of plant material in water affects the seeking behaviour and the acceptance of a potential breeding site by gravid females, likely because it may inform about the presence of nutrients [28]. Several chemical compounds from plant material have already been isolated and characterized (For review see [28]). Most importantly, the attractiveness of a site, driven by the presence of either conspecifics, heterospecifics or plant material, is expected to be closely associated with bacterial growth, as demonstrated by the increased attractiveness of oviposition site waters when fermented [20, 57], as well as reduced attractiveness when sterilized or treated with antibiotics [54, 58]. This suggests that the process of bacterial growth and activity, perceived by gravid mosquitoes through secondary metabolite production [29], likely indicates a highly nutritive medium.

### Compounds mediating oviposition for which only lab evidences is available

An “oviposition attractant” is a substance that causes gravid females to make an oriented flight toward the oviposition substrate (conversely to “repellent”) while an “oviposition stimulant” is a substance that elicits the oviposition behaviour after landing on the substrate (conversely to “deterrent”) [59]. Behavioural assays performed under laboratory conditions are conducted using the two-choice bioassay described previously, and do not allow the separation of short-range (stimulant/deterrent) from long-range (attractant/repellent) cues.

### Attractants and stimulants

Some compounds previously identified in the chemical signature of eggs were tested for their effect on oviposition under laboratory experiments (Table 1). Among them, dodecanoic acid, Z-9-hexadecenoic acid, hexadecanoic acid, tetradecanoic acid and caproic acid showed significant oviposition stimulation, with OAI values of 0.54 at 100 ppm for dodecanoic acid, 0.55 at both 10 and 100 ppm for Z-9-hexadecenoic acid, 0.40 at 1 ppm for hexadecanoic acid, 0.65 at 10 ppm for tetradecanoic acid, and 0.32 at 1 ppm for caproic acid [46, 47, 60]. In the same way, four long-chain fatty acids identified in larval extracts, pentadecanoic acid, tetradecanoic acid, myristoleic acid and isovaleric acid, were assayed in laboratory settings. Pentadecanoic acid and the blend of compounds at a ratio mimicking the one observed in larvae induced significant stimulation, with OAI values of 0.38 at 10 ppm for pentadecanoic acid and 0.21 at 1 ppm for the blend. Interestingly, these candidates were as efficient for inducing oviposition as the water that previously contained larvae [61].

**Table 1:** Candidates that elicited significant oviposition stimulation or deterrence in laboratory assays and level of validation

Candidate	Effect on oviposition	OAI	Dose^a^	Validation in field experiments	References
Crude semiochemicals of plant origin					
Infusions					
Graminea (*Panicum maximum*) infusion	Stimulation	NM	10%	Yes	[57]
Bermuda grass (*Cynodon dactylon)* infusion	Stimulation	0.5	5% and 10% infusion	NT	[62]
Cashew leaves (*Anacardium occidentale)* infusion	Stimulation	NM	50%	NT	[65]
Tea infusion	Stimulation	NM	3 teabags or 4 capsules in 8 l of water	NT	[35]
Bamboo leaf (*Arundinaria gigantea*) and white oak leaf (*Quercus alba*) infusion	Stimulation	NM	50%	NT	[88]
Neem seed kernel (*Azadirachta indica*) infusion	Deterrence	−0.6	10%	NT	[62]
Dried tobacco leaves (*Nicotiana* spp.) infusion	Deterrence	−0.7	10%	NT	[62]
Extracts					
Beetroot (*Beta vulgaris*) peel	Stimulation	0.7	10 g in 600 ml of water	Yes	[33]
Sweet wormwood (*Artemisia annua)* extracts	Deterrence	−0.94	500 ppm	NT	[73]
Essential oils					
Peppermint oil, basil oil, rosemary oil, citronella oil and celery seed oil	Deterrence	−0.22 to −0.95	10%	NT	[68]
Crude semiochemicals of larval origin					
Larvae infusion	Stimulation	0.5	100 larvae/100 ml	Yes	[41, 61]
Larvae infusion	Deterrence	−0.45	5 larvae/2 ml		[42]
Chemical compounds					
* n*-Heneicosane	Stimulation	0.09	10 ppm	Yes	[30, 64, 87]
Geosmin	Stimulation	0.2	0.01%	Yes	[33]
Tetradecanoic acid	Stimulation	NM	10 ng	Yes, in blend with nonanoic acid and tetradecanoic acid methyl ester	[88]
Nonanoic acid	Stimulation	NM	100 ng	Yes, in blend with tetradecanoic acid and tetradecanoic acid methyl ester	[88]
Tetradecanoic acid methyl ester	Stimulation	NM	10 ng	Yes, in blend with tetradecanoic acid and nonanoic acid	[88]
Nonanal	Stimulation	NM	NM	Yes	[11, 12, 31]
Dodecanoic acid	Stimulation	0.54	100 ppm	NT	[47]
Z-9-Hexadecenoic acid	Stimulation	0.55	10 and 100 ppm	NT	[47]
Caproic acid	Stimulation	0.32	1 ppm	NT	[46]
Pentadecanoic acid	Stimulation	0.38	10 ppm	NT	[61]
Myristoleic acid	Deterrence	−0.7	100 ppm	NT	[61]
Skatole	Stimulation	0.41	500 ppm	NT	[64]
Skatole	Deterrence	NM	100 μg/l	NT	[76]
* p*-Cresol	Stimulation	0.23	100 ppm	NT	[64]
* p*-Cresol	Deterrence	−0.4 to −1	10^–8^ to 10^3^ ppm	NT	[64, 74]
Phenol	Stimulation	0.16	50 ppm	NT	[64]
Propyl octadecanoate	Stimulation	0.43	10 ppm	NT	[66]
Methyl dodecanoate	Deterrence	−0.92	100 ppm	NT	[47]
Methyl (Z)-9- hexadecenoate	Deterrence	−0.86	100 ppm	NT	[47]
Tetradecyl heptanoate	Deterrence	−0.81	10 ppm	NT	[66]
Blend of chemical compounds					
Blend of p-cresol, skatole, phenol, n-heneicosane	Stimulation	0.5	Skatole at 500 ppm, p-cresol at 100 ppm, phenol at 50 ppm, n-heneicosane at 10 ppm	NT	[64]
Blend of nonanal, decanal, skatole	Stimulation	NM	NM	Yes	[11, 12, 31]
Blend of nonanal, decanal, *p*-cresol	Stimulation	NM	NM	Yes	[11, 12, 31]
Blend of pentadecanoic acid, myristoleic acid, myristic acid, isovaleric acid	Stimulation	0.21	1 ppm	NT	[61]
Blend of pentadecanoic acid, myristoleic acid, myristic acid, isovaleric acid	Deterrence	−0.65	100 ppm	NT	[61]
Blend of nonanoic acid, tetradecanoic acid and tetradecanoic acid methyl ester	Stimulation	NM	10^–4^ and 10^–5^ ppm	Yes	[32]

OAI is calculated as (NT – NS)/(NT + NS), where NT and NS denote the mean number of eggs laid in the treatment and control, respectively. Positive OAI values indicate oviposition stimulation/attraction, whereas negative values indicate repellence/deterrence

*NT* not tested, *NM* not mentioned

^a^Only the doses that elicited the most significant stimulation/attractance or deterrence/repellence are presented here

Plant infusions have also been frequently investigated for the development of odour-bated traps because of their low implementation cost. Fortunately, laboratory assays have shown their potential in mediating oviposition in gravid *Ae. aegypti* females. Indeed, the infusion of Bermuda grass (*Cynodon dactylon*) collected more eggs than untreated water when presented to mosquitoes in 5% and 10% solutions compared with the control, with an OAI value of 0.55 [62]. For this infusion, the chemical signature has been investigated and involved 5 compounds: skatole, *p*-cresol, 4-ethylphenol, phenol, and indole [63]. Among them, skatole, *p*-cresol and phenol outperformed water in stimulating egg-laying when presented separately, with OAI values of 0.41 at 500 ppm for skatole, 0.23 at 100 ppm for *p*-cresol, and 0.16 at 50 ppm for phenol. When presented in a blend using these doses in a mixture with the oviposition pheromone *n*-heneicosane at 10 ppm, the chemicals showed a synergistic effect, receiving 67% of the total eggs compared with the control, with an OAI of 0.50 [64]. Then, infusion of cashew leaves (*Anacardium occidentale*) was also shown to be a good oviposition stimulant, collecting significantly more eggs than did water when diluted at 50% (OAI not given) [65]. Finally, several synthetic esters were also tested for their effect on oviposition. Among them, propyl octadecanoate achieved a significantly higher oviposition response than did water, with an OAI of 0.43 at 10 ppm [66]. Despite the potential of all these compounds, complementary studies such as specificity studies and field experiments are lacking to validate their use in vector control programmes.

### Repellents and deterrents

Gravid females can also detect oviposition deterrents that may be toxic to larvae or indicate overcrowding, and avoid oviposition in those sites [67] (Table 1). In this context, some compounds originating from mosquito immature stages were tested for their potential in deterring oviposition. First, myristoleic acid, a long-chain fatty acid identified in larval extracts, induced high oviposition deterrence, with an OAI of −0.70 at 100 ppm [61]. Interestingly, all the esters identified in mosquito eggs, such as methyl dodecanoate and methyl (Z)-9-hexadecenoate, were deterrents for females, with OAI values of −0.92 and −0.86, respectively, at 100 ppm [47]. In agreement with these observations, many synthetic esters of plant origin elicited significant oviposition deterrence compared with water. For instance, tetradecyl heptanoate showed OAI values of −0.81 at 10 ppm [66]. The presence of esters is thought to be associated with overcrowding, indicating a non-suitable site [47]. In the same way, essential oils such as peppermint oil, basil oil, rosemary oil, citronella oil and celery seed oil were tested for their properties and also induced oviposition deterrence (OAI of −0.72 for 0.1% peppermint oil for instance), most probably due to the presence of monoterpenoids, known for their repellent, antifeedant and insecticidal properties against insects [68–70].

Extracts and infusions from several plant tissues have also been tested for their potential in deterring oviposition and some of them (*Bryopsis pennata*, *Syzygium lanceolatum*) showed no toxicity against non-target organisms, including mosquito predators [71, 72]. Solvent-extracted cells of sweet wormwood (*Artemisia annua*) showed excellent deterrent potential, with negative OAI values ranging from −0.21 at 50 ppm to −0.94 at 500 ppm [73]. Also, infusions of neem seed kernel (*Azadirachta indica*) and dried tobacco leaves (*Nicotiana* spp.) showed OAIs of −0.60 and −0.70 when dosed at 10% [62].

The deterrent/repellent effect of certain compounds on the oviposition behaviour may follow a dose-dependent response, where the repellence increases with higher doses, as can be observed with several fatty acid esters [66]. This might be explained by increased toxicity of such products when they are present in high concentrations within breeding sites. Interestingly, this observation can be reversed with certain molecules: whereas *p*-cresol and skatole were documented as stimulants by some authors when tested at a dose of 100 ppm [64], in other studies they were observed to be deterrents at doses ranging from 10^–8^ to 10^3^ ppm for *p*-cresol [74, 75] and at 10^–1^ ppm for skatole [76], using the same methodology. Along with these results, different OAIs can be observed among several studies when similar compounds are tested at the same dose. For instance, OAI values for tetradecanoic acid tested at 10 ppm observed were 0.48 [47], 0.65 [60] and −0.04 [61]. The recorded OAI values for hexadecanoic acid tested at 100 ppm were −0.062 [46] and −0.43 [47], and the values for (Z)-9-hexadecenoic acid tested at 10 ppm were −0.1 [46] and 0.55 [47]. All these observations suggest that the observed effect of a chemical on oviposition in laboratory settings is strongly dependent upon the tested dose but also upon complex factors that could be experimental conditions which may or may not allow its volatilization, solvent used, mosquito genotype and experience, and the duration of the assay. In view of the potential of these extracts, further studies are needed to standardize and finely determine the conditions guaranteeing an implementation success in integrated vector management programmes. This is essential for endemic areas for mosquito-borne diseases, where many aquatic breeding sites cannot be removed, drained, or filled for several reasons. For these water collections, the use of oviposition repellents into the water, when properly dosed and the remanence assessed, could help to reduce the infestation rates, as it was already proposed with larvicides [77]. Following the principles of push–pull strategies relatively common in integrated pest management, oviposition deterrents could also be used to deter gravid females and redirect them towards an attractive lethal gravitrap [78–80]. Such a strategy may reduce larvicide costs by providing information about localization of breeding sites that should be given priority for treatment in larval source management programmes [81].

### Compounds mediating oviposition with field validation

The behavioural validation of chemical candidates implies the use of traps that measure either the number of mosquitoes collected or the number of eggs received, and can be implemented in semi-field experiments (i.e. controlled chambers or insect-proof greenhouses) and/or directly in field surveys (i.e*.* at the household and community scale). Several studies have performed field assays to test the efficacy of candidates previously identified as oviposition stimulants in laboratory experiments (Table 1). Regarding the chemical signature of *Ae. aegypti* immature stages, only *n-*heneicosane, the compound isolated from the larval cuticle, has received attention with both lab and field assays. In laboratory settings, it has been shown to be a good stimulant for egg-laying compared with water when dosed at 10 ppm (OAI of 0.09) and 69 ppm (OAI of 0.28) [48, 64]. Then, field experiments were conducted across several localities in India for evaluating the ability of containers containing *n*-heneicosane (15.10^4^ ppm) plus a growth regulator (diflubenzuron, NeemAzal or triflumuron) to trap and inhibit the growth of *Ae. aegypti* and *Ae. albopictus* mosquitoes. The results of the study evidenced a higher number of eggs collected in experimental containers than in control ones, with 73% of eggs deposited in treated containers in the site of Bengaluru where *Ae. aegypti* is present (OAI of 0.45). This effect was however not significant in Delhi, another study area (OAI of 0.1). An inhibition of pupation was also recorded in treated containers in both sites [30]. The formulation of *n*-heneicosane plus a growth regulator then seems to be a good candidate for the development of traps against *Ae. aegypti* mosquitoes. In this context, a patent with the formulation has been deposited and several toxicology assays were performed for evaluating its safety [82, 83], rendering *n*-heneicosane the most advanced compound of larval origin in the process of validation. However, as a main drawback, this compound has also been documented in the cuticle of other arthropod species [84, 85] as well as in plants [86], suggesting that it might not be involved in the species-specific signature of *Ae. aegypti* and that it might also induce a behavioural response in other arthropod species. In mosquitoes, *n*-heneicosane has also been found in the cuticle of *Ae. albopictus* and induced stimulation of egg-laying when dosed between 0.1 and 10 ppm (OAI of 0.2 at 1 ppm) [87]. While this common behavioural induction may represent a beneficial approach for trapping both species, additional studies remain necessary to assess the specificity of this candidate to *Ae. aegypti* or more generally to mosquito pests, as well as its impact on non-target arthropod species. Then, regarding traps using attractants of plant origin, fermented infusion of graminea *Panicum maximum* collected significantly more eggs than did water when tested in two-choice laboratory assays and dosed at 50% (OAI not given) [65]. A similar effect was observed in field experiments conducted in Brazil, where traps baited with 10% fermented infusion of *P. maximum* showed 47% of positivity for *Ae. aegypti,* against 21% for the control water [57]. Chemical identification of fermented infusion of *P. maximum* provided evidence of seven different compounds: nonanal, decanal, benzothiazole, skatole, *p*-cresol, limonene, and indole [31]. An oviposition trap constituted from the aldehyde nonanal have been developed under the patent AtrAedes^®^ for the surveillance of *Ae. aegypti*, under the name MosquiTRAP™ (Ecovec Inc.) [11, 12]. Further studies showed that the efficacy of the trap could be increased when a blend of either decanal and skatole or decanal and *p*-cresol was added to the attractant, both in semi-field and field experiments, highlighting the synergistic effects between compounds and improving the AtrAedes^®^ mixture [31]. Then, 1-week-old infusions of bamboo leaf (*Arundinaria gigantea*) and white oak leaf (*Quercus alba*) strongly stimulated egg-laying in the laboratory, receiving up to 84% of total eggs (OAI not given) [88]. When tested in laboratory settings, a trap baited with 2–4-week-old infusion of oak leaves (*Quercus* spp.) consistently caught on average more than 80% of all gravid females at their first egg-laying attempt [35], suggesting an attractive rather than a stimulant oviposition signal. Bioactive compounds belonging to white oak leaves were identified as being of bacterial origin and consisted of a mix of carboxylic acids ranging from nonanoic acid to octadecanoic acid, and several carboxylic acid methyl esters [88, 89]. Synthetic compounds were assayed both individually and in blend in laboratory experiments. A blend composed of 83% tetradecanoic acid, 16% nonanoic acid and 1% tetradecanoic acid methyl ester elicited strong oviposition stimulation, with 85% of the total eggs laid in a solution containing 3.10^−4^ ppm of the blend (OAI of 0.7) [88]. Experiments conducted in a dengue endemic area in Brazil confirmed this effect, both using semi-field and field experiments, and evidenced twice more eggs laid in traps baited with the mixture at doses of 3.10^−4^ and 3.10^−5^ ppm than in the control [32]. Finally, field experiments conducted in Florida showed that ovitraps baited with geosmin, a volatile organic compound previously identified in cyanobacteria and showing attractive properties in laboratory assays, held an increased number of eggs relative to control traps, with a mean of 35 eggs in traps baited with synthetic geosmin dosed at 0.1% against 26 eggs in the control (OAI around 0.2). Interestingly, the stimulant properties were enhanced when geosmin was replaced with peel extracts of beetroot (*Beta vulgaris*), a natural source of geosmin (OAI around 0.7), which highlights its potential to be used as a cheap ovitrap against *Ae. aegypti* [33].

### Compounds whose mode of sensory perception is known

Despite the amount of documentation about the ability of some organic compounds to mediate oviposition in *Ae. aegypti*, their sensory perception is rarely assessed. Consequently, whether these compounds act through the olfactory or gustatory pathway, or both, is still not known. Methods for assessing the distance of action of these compounds involve modified two-choice assays using sticky screen cups to which mosquitoes could be trapped when they fly over the substrate [90], dual-choice oviposition assays without contact with the tested chemical [50], behavioural assays in an olfactometer, and electrophysiology assays (electroantennography [EAG], single-sensillum recording). Other techniques such as calcium imaging and CRISPR/Cas9 can also be used to inform about sensory perception, although, to the best of our knowledge, they were not used to depict the mechanisms of the candidates presented in this review. So far, among the candidates previously observed to be oviposition stimulants, some have been shown to be directly involved in the olfactory perception. First, *n*-heneicosane, the pheromone identified in the larval cuticle and validated in laboratory and field settings, induced significant antennal responses in gravid females during EAG assays [87, 91]. In behavioural assays, this compound has been shown to act both as an attractant and stimulant [91]. Then, among the fatty acids identified in larval extracts, pentadecanoic, tetradecanoic and myristoleic acids did not elicit antennal detection, suggesting that they might act as tactile cues [61]. Similar to the results obtained by Ponnusamy et al. and Barbosa et al., who used tetradecanoic acid as the main compound of their attractive blend [32, 88], it can be suggested that this compound may not act as a volatile cue but rather may stimulate oviposition once females land on the water surface. Additionally, chemicals identified in fermented infusion of *P. maximum*, nonanal, decanal, benzothiazole, skatole, *p*-cresol, limonene, and indole, were tested and induced significant EAG detection at concentrations of 10^2^ to 10^6^ ng [31]. Then, the sticky-screen bioassay method showed that white oak and bamboo leaf infusions (from which tetradecanoic acid, nonanoic acid and tetradecanoic acid methyl ester were identified as active compounds) induced attractance without contact [90]. Geosmin, a cyanobacteria-produced volatile, also elicited antennal detection in EAG assays [33]. Finally, the fatty acid ester propyl octadecanoate, previously identified as a stimulant for egg-laying in laboratory bioassays, elicited EAG responses and showed attractance to *Ae. aegypti* females in an olfactometer at doses of 10^−6^ and 10^−5^ ppm [92]. Several of these candidates seem to act as olfactory cues and are promising for the development of odour-based traps due to their long range of action. Candidates evidenced as tactile cues could be assayed together with volatile cues in traps to look for a potential synergistic effect.

## Remaining challenges for the development of specific traps against gravid *Ae. aegypti*

### Critical analysis and proposed framework for complete validation of a chemical candidate

Although laboratory assays have provided a panel of potential candidates for trap development (stimulating or deterring oviposition), many of them remain to be validated under field settings. Importantly, to the best of our knowledge, so far no oviposition deterrent has been tested further than in laboratory settings. Also, whereas many studies have focused on measuring oviposition behaviour, studies aiming at characterizing the chemical profile of the candidates and their mode of detection are still scarce. Additional studies are then necessary in order to (i) validate the candidates identified, (ii) characterize the bioactive compounds within natural extracts, and (iii) optimize the efficacy and the specificity by combining attractants/stimulants and repellents/deterrents, and by integrating both long-distance and contact cues within the traps.

As an important feature, most of the advanced candidates in the validation process are stimulants of plant origin. Although they represent cost-effective options, their potential in inducing behavioural effects in non-target organisms is not known. However, when tested in laboratory experiments, plant infusions such as Bermuda grass, neem seed kernel and tobacco leaves induced the same effect on the oviposition in several mosquito species (*Ae. aegypti*, *Anopheles stephensi* and *Culex quinquefasciatus*) [62], suggesting that they also interact with the behaviour of other arthropods. Also, ovitraps baited with plant material have been shown to induce much bycatch of arthropods [23]. These observations raise questions about the specificity of plant material against mosquitoes, and highlight the need for testing the candidates over several arthropod species to guarantee their sustainable use. In this context, the use of pheromones of larval origin would greatly contribute to the development of species-specific traps. As a major obstacle, no volatility has ever been evidenced from *Ae. aegypti* immature stages using headspace sampling methods. Among candidates identified using solvent extraction methods, most of the molecules are long-chain fatty acids and are therefore expected to present low volatility and act as taste cues rather than at distance. Also, *n*-heneicosane remains the only chemical previously identified in the chemical signature of *Ae. aegypti* that has been tested under field experiments. The lack of studies on the chemical signature of immature stages and the validation of oviposition cues in the field strongly limits the panel of candidates available so far. In order to implement highly efficient and specific traps, more candidates should be identified in immature stages and those existing should be brought to the next stage of validation, by evaluating their sensory detection and by assessing their potential in field studies.

**Fig. 1:**
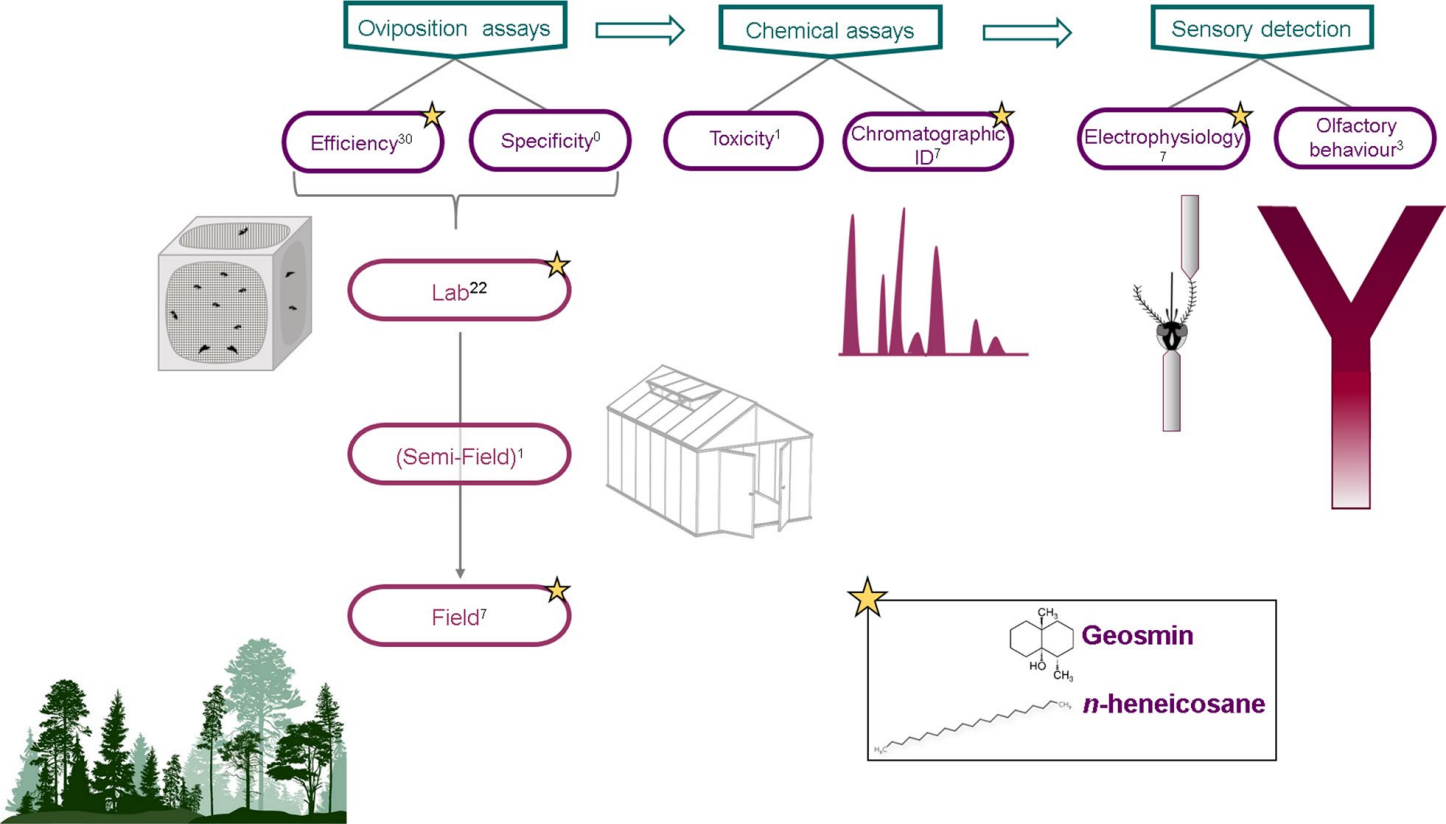
Optimal workflow for validating a chemical candidate to be used in the implementation of gravitraps. The semi-field step presented in brackets indicate a facultative step. The proposed order for chemical and sensory assays is not mandatory. The stars represent the steps validated by the two most advanced candidates, geosmin and *n*-heneicosane. Superscript numbers show the number of candidates (individual compounds or extracts) validated for each step. The literature search covered articles published from 1984 to 2021

With the aim of providing guidelines for operational studies and homogeneity of experimental approaches, we propose a complete framework to follow from the beginning of the study to the final validation of (a) chemical candidate(s). The complete workflow is presented in Fig. 1 and includes the following steps:

- Laboratory oviposition assays under controlled conditions (two- or multiple-choice assays offering different substrates for oviposition)
- Identification of the chemicals involved (through chromatographic analysis)
- Assessment of mosquito sensory detection (smell *versus* taste) using techniques such as olfactometer assays, sticky screen bioassay and electrophysiology techniques (electroantennography or single sensillum recording)
- Oviposition bioassays at a larger scale (i.e. semi-field assays—facultative—and field assays, with the most efficient dose observed in laboratory). After assessment of the efficacy of candidates, the efficacy of selected chemicals should be challenged against existing commercial traps and natural breeding sites
- Specificity assays
- Toxicity assays

We believe that all these parameters, when investigated, would fully depict the potential of a candidate and ensure its incorporation into integrated vector management programmes to guarantee sustainable control of *Ae. aegypti*.

### Identifying research priorities for the validation of the existing candidates

**Fig. 2:**
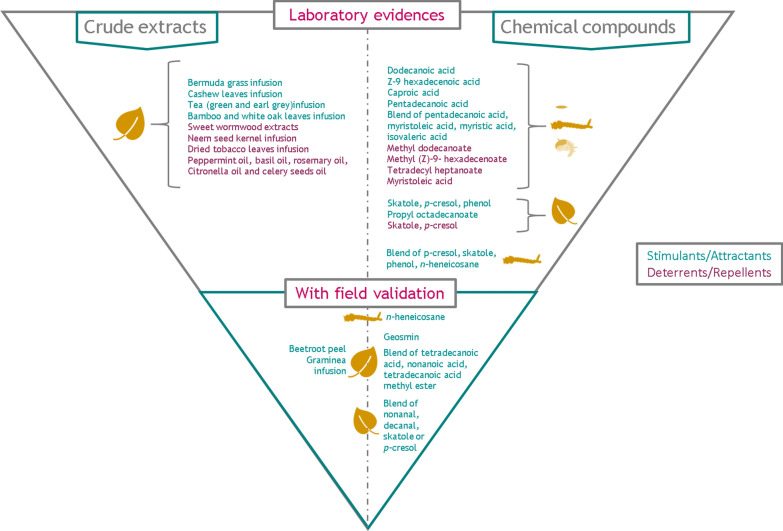
Candidates identified for mediating oviposition and their level of validation. Icons show the origin of the signals: plant or immature stages (eggs, larvae, pupae)

The advances in the validation of the existing candidates are summarized in Fig. 2. Following the proposed framework (Fig. 1), the next steps that need to be performed for the candidates that have received only laboratory validation involve (i) for stimulants: chemical characterization (cashew leaves, tea infusions), assessment of the sensory perception (dodecanoic acid, Z-9 hexadecenoic acid, caproic acid) and field experiments (tetradecanoic acid, pentadecanoic acid, skatole, p-cresol, propyl octadecanoate, and blend of pentadecanoic, isovaleric, myristoleic and tetradecanoic acids); and (ii) for deterrents: chemical characterization (sweet wormwood, neem seed kernel, dried tobacco leaves, essential oils), sensory assays (methyl dodecanoate and methyl (Z)-9- hexadecenoate, tetradecyl heptanoate) and field experiments (myristoleic acid). Regarding compounds that have received both laboratory and field validation, one candidate, *n*-heneicosane, fulfilled almost all the steps and entered the process of final validation. Three candidates, i.e. the mixture of compounds from *P. maximum*, those from *Quercus* spp., and geosmin, have fulfilled laboratory and field experiments, and their sensory detection has also been investigated. Next steps for validation involve toxicity and specificity assays (evaluation of their influence on non-target organisms) at the doses identified, in order to fully decipher their potential for use in sustainable programmes and, in the future, for developing a controlled-release formulation. Overall, the efficacy of the most advanced candidates should be compared under different contexts, using different mosquito genotypes and environments as a last step for validation. Also, taking into account the effect of mosquito physiology on the efficacy of the trap is essential, as, for instance, infection with viruses may modify oviposition preferences [76].

### Methodological challenges and optimization for better reproducibility

A major obstacle for the generalization of experimental data to natural conditions lies in the methodology that is conventionally used for measuring oviposition behaviour. The proposed protocols, consisting in considering the number of eggs laid as a proxy for female preferences, would be rigorous for mosquitoes that lay all their eggs at the same timing and site, such as *Culex* spp. However, other mosquito species such as *Aedes* spp. tend to lay eggs on different occasions [25, 93] and sites [94], which implies that a female might (i) randomly deposit its eggs among several equal substrates and, (ii) refuse to lay eggs or deposit only a small fraction of these if provided with sub-optimal choices. This is exacerbated by the tendency of *Ae. aegypti* females to avoid ovipositing in sites containing its eggs or eggs from conspecifics [45]. For these species, egg-count should not be used as a direct measure of responding females. However, the total number of eggs is conventionally divided by the total number of released females, whereas it is the behaviour of only a few mosquitoes that is actually tested. In this context, a study using *Anopheles gambiae* showed that this species, when offered similar substrates, distributes 2/3 of its eggs in one substrate and 1/3 in the other (with a significant *P*-value), evidencing that a higher egg batch does not necessarily mean a higher preference [95]. As a consequence, the calculation of the OAI based only on the total number of eggs laid and on the total of released females is not adequate and might both mask data heterogeneity and inter-replicate variability, and lead to erroneous interpretation. It is worth noting that the calculation of the OAI was initially developed for egg rafts of *Culex* mosquitoes [36], and was, subsequently, wrongly extrapolated to *Anopheles* and *Aedes* mosquitoes. This bias is exacerbated when replication is insufficient, resulting in significant artefacts that are more based on stochastic effects than on real differences [95]. Okal et al. [95] and Corbet et al. [96] proposed revised methodologies for assessing oviposition preferences with better robustness and reproducibility, by taking into account skip-oviposition as well as putting a higher focus on statistical relevance. In agreement with these authors, we recommend, first, including only responders in the dataset, by either (i) performing bioassays using individual females only, with the drawbacks that this represents logistical and timing constraints and is not applicable in semi-field assays, or (ii) dissecting female ovaries to confirm the presence or absence of eggs and to ascertain the percentage of responders. Then, to cope with the heterogeneity in egg distribution, the authors highlight the importance of offering to females a non-binary choice, by separating the tested compound and the control into several different cups. When possible, the number of females visiting the concerned oviposition substrates could also be recorded. Their trajectory and the time before egg-laying are parameters that can also be recorded using imaging cameras, which will help differentiate long- and short-range cues [97]. For providing the methodological choices with a mathematical basis, we encourage the preliminary use of power calculation (available in R software [98], “power.prop.test” function) to determine the group size necessary to obtain a dataset that allows for statistical comparisons with sufficient power. For more data robustness and quality, we also encourage authors to present the OAI together with data about the proportion of eggs laid between substrates. This latter should be analysed with generalized linear models with binomial distribution, and the associated *P*-value should be used to accept the test as significant, rather than the value of the OAI.

When performing the first laboratory bioassays, the efficacy of a candidate should be evaluated in a wild colony, by performing dose-responses. However, most of the compounds have reversal valence for mosquitoes, meaning that they can have both deterrent effect at high doses and attractant effect at low doses. The choice of the tested concentrations is essential, as the effect of a given concentration may also differ between laboratory and field conditions. Also, behavioural experiments are often performed with concentrations that are not biologically relevant (sometimes up to 500 ppm). Indeed, compounds are usually active at lower concentrations, as some chemicals are capable of inducing a behavioural response at the nanogram level [88]. The lowest dose that induces a significant behavioural response should be considered, to increase the possibility of use in the field, both in terms of cost and toxicological impact. However, depending on the experimental context (e.g. cage vs semi-field bioassays), the dose tested may not have the same influence on the mosquito response, due to several factors such as the “activation distance”, volatilization, or competition with other environmental stimuli. Also, to guarantee sustainability, the testing of blends should be prioritized, because their use would enable delay of habituation and behavioural avoidance, as observed for semiochemical control methods against orchard pests [99].

## Conclusion

We believe that the framework proposed in this review, enriched with the methodological updates and recommendations, would enable the revelation of female substrate preferences unambiguously, with reproducible and statistically powerful results, which is essential for the implementation of effective and species-specific odour-based traps directed against gravid *Ae. aegypti* females.

## Data Availability

Not applicable.
